# Positive end-expiratory pressure affects the value of intra-abdominal pressure in acute lung injury/acute respiratory distress syndrome patients: a pilot study

**DOI:** 10.1186/cc9193

**Published:** 2010-07-21

**Authors:** Daniel Verzilli, Jean-Michel Constantin, Mustapha Sebbane, Gérald Chanques, Boris Jung, Pierre-François Perrigault, Manu Malbrain, Samir Jaber

**Affiliations:** 1 Intensive Care and Transplantation Unit, Department of Anaesthesiology and Critical Care, University Saint Eloi Hospital, 80, avenue Augustin Fliche, University of Montpellier I, F-34295 Montpellier Cedex 5, France; 2 Department of Anesthesiology and Critical Care medicine, Hôtel-Dieu Hospital, University Hospital of Clermont-Ferrand, F-63058 Clermont-Ferrand, France; 3 Department of Intensive Care Medicine, Ziekenhuis Netwerk Antwerpen, Campus Stuivenberg, Lange Beeldekensstraat 267, 2060, Antwerpen 6, Belgium

## Abstract

**Introduction:**

To examine the effects of positive end-expiratory pressure (PEEP) on intra-abdominal pressure (IAP) in patients with acute lung injury (ALI).

**Methods:**

Thirty sedated and mechanically ventilated patients with ALI or acute respiratory distress syndrome (ARDS) admitted to a sixteen-bed surgical medical ICU were included. All patients were studied with sequentially increasing PEEP (0, 6 and 12 cmH_2_O) during a PEEP-trial.

**Results:**

Age was 55 ± 17 years, weight was 70 ± 17 kg, SAPS II was 44 ± 14 and Pa_O2_/F_IO2 _was 192 ± 53 mmHg. The IAP was 12 ± 5 mmHg at PEEP 0 (zero end-expiratory pressure, ZEEP), 13 ± 5 mmHg at PEEP 6 and 15 ± 6 mmHg at PEEP 12 (*P *< 0.05 vs ZEEP). In the patients with intra-abdominal hypertension defined as IAP ≥ 12 mmHg (*n *= 15), IAP significantly increased from 15 ± 3 mmHg at ZEEP to 20 ± 3 mmHg at PEEP 12 (*P *< 0.01). Whereas in the patients with IAP < 12 mmHg (*n *= 15), IAP did not significantly change from ZEEP to PEEP 12 (8 ± 2 vs 10 ± 3 mmHg). In the 13 patients in whom cardiac output was measured, increase in PEEP from 0 to 12 cmH_2_O did not significantly change cardiac output, nor in the 8 out of 15 patients of the high-IAP group. The observed effects were similar in both ALI (*n *= 17) and ARDS (*n *= 13) patients.

**Conclusions:**

PEEP is a contributing factor that impacts IAP values. It seems necessary to take into account the level of PEEP whilst interpreting IAP values in patients under mechanical ventilation.

## Introduction

Patients with primary acute lung injury (ALI) or acute respiratory distress syndrome (ARDS) may develop secondary abdominal pathologies associated with increased intra-abdominal pressure (IAP) and, vice versa, primary abdominal problems can be associated with intra-abdominal hypertension (IAH) and secondary ALI/ARDS [1-3]. Correct bedside measurement of IAP in daily clinical practice is important. The IAP value is influenced by physiological (eg, body weight, body position, abdominal muscle activity), non-physiological (eg, surgical pneumoperitoneum) and multiple pathological situations (eg, abdominal trauma, pancreatitis, liver transplantation) [2,4]. A persistent increase of IAP can produce multiple adverse effects, involving both intra-abdominal (eg, kidney, bowel) and extra-abdominal (eg, respiratory, cardiovascular) organ systems. Clinical symptoms can occur when the IAP exceeds 12 mmHg [5]. The management of abdominal compartment syndrome (ACS) consists of optimising medical treatment; however, if that fails, surgical decompression should be considered, because it can be a life-saving procedure. Medical management of ALI/ARDS patients with ACS can consist of sedation (sometimes with the use of neuromuscular blockers), intubation and mechanical ventilation with positive end-expiratory pressure (PEEP), sometimes at high levels [5]. Several studies focused on the effects of IAH on other organ systems, especially the respiratory system [1,5]. The World Society of the Abdominal Compartment Syndrome (WSACS) [6] has published consensus definitions and guidelines for the diagnosis, management, prevention and treatment of IAH and ACS [5]. Although methods used to measure IAP are now well defined, few studies [7-9] have investigated the influence of the adjustment of respiratory parameters on IAP values. We hypothesised that an increase in the PEEP level can lead to an increase in IAP values in ALI/ARDS patients. The aim of this physiological pilot study was to evaluate the impact of different PEEP levels on IAP values.

## Materials and methods

Over a six-month period (January to June 2006) all consecutive patients admitted to the ICU of Saint-Eloi University hospital in Montpellier, France, with abdominal diseases (peritonitis, pancreatitis, liver transplantation and abdominal trauma) that had IAP monitoring and that were under mechanical ventilation for ALI/ARDS (partial pressure of arterial oxygen (PaO_2_)/fraction of inspired oxygen (FiO_2_) < 300 mmHg) were included. The ventilation modality used was volume controlled and the tidal volume selected for each patient was unchanged throughout the whole period of the study. All patients were sedated to obtain a Richmond Agitation Sedation Scale (RASS) at -5 or -4 without muscle relaxant. Demographic data were also recorded.

The protocol was approved by the local ethics committee and informed consent was provided by patients or next of kin. Bedside measurements of IAP were performed by transduction of pressure from an indwelling bladder catheter after priming with sterile saline (50 ml), according to the modified Kron technique [10]. IAP was always measured in the complete supine position at end-expiration with the transducer zeroed at the midaxillary line (at the level of the iliac crest), in stable conditions (absence of abdominal muscle contractions). Together with IAP, hemodynamic and respiratory parameters were measured during a PEEP trial with three consecutive PEEP settings in the same order (0, 6 and 12 cmH_2_O). These measurements were obtained after a stabilisation period of five minutes after change in PEEP setting.

Results are expressed as mean ± standard deviation. Patients with an IAP below 12 mmHg (low-IAP group) were compared with those with an IAP of 12 mmHg or more (high-IAP group) at zero end-expiratory pressure (ZEEP). The appropriate analyses of variance were applied for all comparisons between the different PEEP levels. A *P *< 0.05 was considered to be significant.

## Results

During the study period, 287 patients were admitted to our ICU. Fourteen women and sixteen men were enrolled in the study. For the whole group, age was 55 ± 17 years, weight was 70 ± 17 kg, height was 167 ± 15 cm, body mass index was 25.2 ± 5.6 kg/m^2^, simplified acute physiology score II was 44 ± 14 and PaO_2_/FIO_2 _ratio was 192 ± 53 mmHg. Main causes of admission were peritonitis (*n *= 19), acute pancreatitis (*n *= 5), haemoperitoneum (*n *= 4) and ileus (*n *= 2).

**Figure 1:**
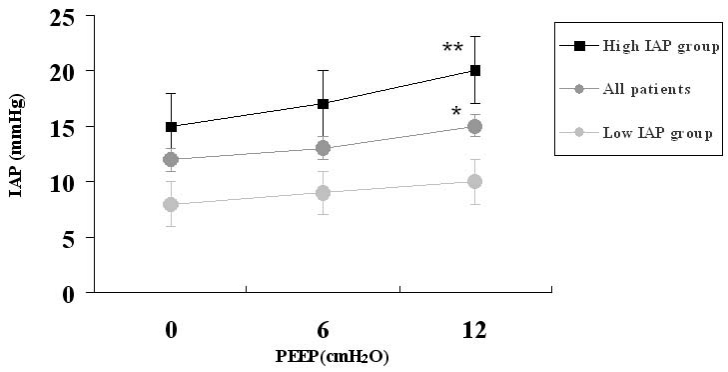
**Effects of PEEP on IAP measure in all patients (*n *= 30), low IAP group (*n *= 15) and high IAP group (*n *= 15)**. *P < 0.05 PEEP0 *vs *PEEP12. **P < 0.01 PEEP0 *vs *PEEP12. IAP, intra-abdominal pressure; PEEP, positive end-expiratory pressure.

Main haemodynamic and respiratory parameters in the low-IAP group (*n *= 15) and the high-IAP group (*n *= 15) are listed in Table 1. No significant difference was observed between the low-IAP and high-IAP group with regard to demographic data. The effects of PEEP on IAP are represented in Figure 1. Increase in PEEP involved a significant rise in IAP (ZEEP vs PEEP 12 cmH_2_O, *P *< 0.05). The overall mean increase in IAP (ΔIAP) was 3.5 ± 1.7 mmHg. This increase was less pronounced when basic IAP (at ZEEP) was less than 12 mmHg (low-IAP group) whereas it was significantly higher at higher baseline levels of IAP (high-IAP group).

**Table 1:** Haemodynamic and respiratory parameters at the three PEEP levels

**PEEP level (cmH**_**2**_**O)**		0	6	12
Heart rate, beats/min	Total (*n *= 30)	87 ± 16	83 ± 22	90 ± 19
	High IAP group (*n *= 15)	95 ± 17	88 ± 27	97 ± 19
	Low IAP group (*n *= 15)	77 ± 10	78 ± 11	80 ± 13
Mean arterial pressure, mmHg	Total (*n *= 30)	89 ± 14	88 ± 14	85 ± 12
	High IAP group (*n *= 15)	90 ± 15	88 ± 15	84 ± 12
	Low IAP group (*n *= 15)	89 ± 12	88 ± 13	87 ± 13
Abdominal perfusion pressure, mmHg	Total (*n *= 30)	77 ± 13	74 ± 14	70 ± 12*
	High IAP group (*n *= 15)	74 ± 15	70 ± 15	64 ± 11*
	Low IAP group (*n *= 15)	79 ± 11	78 ± 12	76 ± 11
Central venous pressure, mmHg	Total (*n *= 30)	11 ± 4	12 ± 4	14 ± 5*
	High IAP group (*n *= 15)	11 ± 4	13 ± 4	14 ± 4*
	Low IAP group (*n *= 15)	10 ± 5	11 ± 5	13 ± 5*
Cardiac output, L/min	Total (*n *= 13)	6.0 ± 2.0	5.8 ± 1.7	5.8 ± 1.8
	High IAP group (*n *= 8)	6.7 ± 2.0	6.4 ± 1.7	6.4 ± 1.8
	Low IAP group (*n *= 5)	5.0 ± 1.6	4.9 ± 1.4	4.7 ± 1.3
Respiratory rate, breaths/min	Total (*n *= 30)	17 ± 4	17 ± 4	17 ± 4
	High IAP group (*n *= 15)	18 ± 4	18 ± 4	18 ± 4
	Low IAP group (*n *= 15)	16 ± 3	16 ± 3	16 ± 3
				
Tidal volume, ml	Total (*n *= 30)	634 ± 142	633 ± 145	629 ± 146
	High IAP group (*n *= 15)	639 ± 178	640 ± 180	634 ± 183
	Low IAP group (*n *= 15)	628 ± 84	624 ± 88	623 ± 89
Plateau airway pressure, cmH_2_O	Total (*n *= 30)	20 ± 5	24 ± 4	29 ± 5*
	High IAP group (*n *= 15)	22 ± 5	25 ± 3	31 ± 3*
	Low IAP group (*n *= 15)	18 ± 5	22 ± 4	26 ± 5*
Dynamic compliance, ml/cmH_2_O	Total (*n *= 30)	41 ± 14	44 ± 15	47 ± 17*
	High IAP group (*n *= 15)	42 ± 17	44 ± 18	46 ± 20*
	Low IAP group (*n *= 15)	40 ± 11	44 ± 12	48 ± 12*

Values are expressed as mean ± standard deviation.

Abdominal perfusion pressure was calculated as: mean arterial pressure minus IAP.

*P < 0.05 PEEP0 *vs *PEEP12.

IAP, intra-abdominal pressure; PEEP, positive end-expiratory pressure.

In the 13 patients in whom cardiac output was measured, an increase in PEEP from 0 to 12 cmH_2_O did not significantly change cardiac output, nor in the 8 of 15 patients of the high-IAP group (Table 1).

The observed effects were similar in both ALI (*n *= 17) and ARDS (*n *= 13) patients.

## Discussion

The main findings of the study are that: moderate PEEP levels can lead to increases in IAP due to transmission of intrathoracic pressure to the abdomen; and this effect should be considered in interpreting IAP measurements in selected patients with ALI/ARDS who are on high levels of PEEP and vice versa setting a lower level of PEEP may have a beneficial effect on IAP.

The IAP is considered to be an important physiological parameter in critically ill patients, and IAP monitoring becomes more often a common practice in the ICU [1,5,10]. The impact of increased IAP on respiratory function in the critically ill has been well studied [2,4]. Mechanical ventilation with high PEEP has been shown to decrease splanchnic perfusion [1,5]. Reduction of splanchnic blood flow is limited at PEEP levels below 10 cmH_2_O, but it is more pronounced at PEEP levels of 15 to 20 cmH_2_O. A significant decrease in abdominal perfusion pressure at 12 cmH_2_O of PEEP was observed and this effect was more pronounced in the high-IAP group (Table 1), confirming previous results [11].

The effects of PEEP on IAP values were such that they would increase the IAH grading. A recent classification by WSACS graded IAH as follow: grade I = IAP 12 to 15 mmHg; grade II = IAP 16 to 20 mmHg; grade III = IAP 21 to 25 mmHg; grade IV = IAP above 25 mmHg [5]. ACS is defined as a sustained IAP of more than 20 mmHg that is associated with new organ failure. Therefore, using the thresholds of the WSACS, the application of 12 cmH_2_O PEEP could increase the IAH grading by one grade. For example, in our study, one patient with grade III IAH was classified as having ACS after PEEP 12 cmH_2_O.

In the literature (Table 2) there is some controversy about the effect of mechanical ventilation and the use of PEEP on IAP. The heterogeneity of the observed differences in reported results between the different studies (Table 2) may be explained in part by the differences in the patient populations. Sussman and colleagues [8] was the first to look at the effects of PEEP on IAP and in their experiment increasing PEEP to 15 cmH_2_O did not affect the IAP. However, in the study by Sussman and colleagues [8], 10 of the 15 studied patients had just had laparotomy. However, others [7,9] have found a mild increase in IAP in patients with a baseline IAP below 12 mmHg when increasing PEEP to 15 cmH_2_O (Table 2). Further, in patients with a baseline IAP above 12 mmHg the effect of PEEP seems to be more pronounced [9] as we found in the present study.

**Table 2:** Effect of PEEP on IAP in human studies

	**Sussman and colleagues **[8]	**Gattinoni and colleagues **[7]	**Torquato and colleagues **[9]	Present study
Publication year	1991	1998	2009	2009
Patients (n)	15	21	30	30
IAP zero reference	SP	SP	SP	MA
IAP volume (ml)	50	100	60	50
PEEP baseline (cmH_2_O)	0	0	0	0
PEEP set (cmH_2_O)	15	15	10	12
ΔPEEP (cmH_2_O)	15	15	10	12
ΔPEEP (mmHg)	11	11	7.4	8.8
IAP at baseline (mmHg)	10.8	10.6 ± 6	8.7 ± 4.5	11.7 ± 4.5
IAP at PEEP (mmHg)	11.7	11.8 ± 6.3	12.3 ± 9.6	15.2 ± 5.8
ΔIAP (mmHg)	0.9	1.4 ± 1.3	3.6 ± 2.6	3.5 ± 1.7

Continuous data are reported as mean ± standard deviation.

ΔPEEP = difference between the two extreme PEEP levels.

ΔIAP = IAP recorded at PEEP minus IAP at baseline.

IAP, intra-abdominal pressure; MA, midaxillary line; PEEP, positive end-expiratory pressure; SP, symphysis pubis.

We found that increasing PEEP from 0 to 12 cmH_2_O resulted in a significant decrease in abdominal perfusion pressure (mean arterial pressure minus IAP) in the high IAP compared with the low-IAP group. This may suggest a difference in preload between the two groups. However, we can only speculate on this. The final message is that application of PEEP may have a detrimental effect on abdominal perfusion pressure especially if the patient is already hypovolaemic and/or already has a high baseline IAP.

Two recent studies looked at the effect of head-of-bed positioning on IAP in critically ill intubated patients [12,13]. The authors concluded that the potential contribution of body position in elevating IAP should be considered in patients with moderate to severe IAH or ACS. In the WSACS recommendations, IAP should always be measured in the full supine position. Acute respiratory failure, especially with elevated intrathoracic pressure and diminished abdominal wall compliance is recognised as a risk factor for IAH or ACS. Medical management of patients with IAH and ALI can lead to the decision of mechanical ventilation with PEEP (sometimes at high levels). In the above mentioned, it is difficult to ascertain the real IAP value and the impact on IAH management. Indeed, like increased PEEP, some other conditions such as prone or semi-recumbent positions add an artifactual pressure on IAP that is removed by returning to the reference supine position.

There are limitations to this study. First, the number of patients studied in each group was small. Second, only PEEP levels up to 12 cmH_2_O were studied. We can speculate that the effect of PEEP is dose related and a higher PEEP level (>12 cmH_2_O) may further increase the IAP in critically ill patients. Third, the fact that preload was not well defined could have affected the results. Fourth, ideally pleural pressure should have been measured in order to quantify the respective effects of chest and lung mechanics on the thoracoabdominal transmission of PEEP to IAP. Finally, because the study was conducted before the publication of the consensus definitions a further limitation of the study was the fact that 50 ml of saline were instilled into the bladder.

## Conclusions

Our results suggest that a high PEEP level may be a risk factor for IAH in selected ALI/ARDS patients. Therefore, PEEP should be applied cautiously in IAH patients. The use of limited PEEP, set to counteract the effects of IAP at the level of the diaphragm may have beneficial effects. As suggested by several authors and the WSACS, standardised IAP measurement methods in mechanically ventilated patients, taking into account body position, zero reference, sedation and muscle paralysis and PEEP levels are needed.

## Key messages

• Methods used to measure IAP are now well defined and some factors may influence the interpretation of IAP values.

• In ARDS patients, PEEP is a risk factor for IAH.

• PEEP should be applied cautiously in IAH patients and thus lowering PEEP to an appropriate level may have a beneficial effect on IAP.

• Standardised IAP measurement methods in mechanically ventilated patients, taking into account body position, zero reference, sedation and muscle paralysis and PEEP levels are needed.

## Abbreviations

ACS: abdominal compartment syndrome; ALI: acute lung injury; ARDS: acute respiratory distress syndrome; FiO2: fraction of inspired oxygen; IAH: intra-abdominal hypertension; IAP: intra-abdominal pressure; PaO2: partial pressure of arterial pressure; PEEP: positive end-expiratory pressure; RASS: Richmond Agitation Sedation Scale; WSACS: World Society of the Abdominal Compartment Syndrome; ZEEP: zero end-expiratory pressure.

## Competing interests

The authors declare that they have no competing interests.

## Authors' contributions

DV and J-MC conducted the research, collected, analysed and performed the statistical analysis. MS, GC, BJ and P-FP made substantial contributions to the conception and design of the study and approved the final version of the manuscript. MM and SJ designed and supervised the research, analysed and interpreted the data, drafted and revised the manuscript. All authors read and approved the final manuscript.
